# Development and Characterization of Self-Adhesive Polymeric Films with Antiallergic Effect

**DOI:** 10.3390/polym17131867

**Published:** 2025-07-03

**Authors:** Ioana Savencu, Sonia Iurian, Cătălina Bogdan, Valentin Toma, Rareș Știufiuc, Ioan Tomuță

**Affiliations:** 1 Department of Pharmaceutical Technology and Biopharmacy, Faculty of Pharmacy, “Iuliu Hațieganu” University of Medicine and Pharmacy, 400012 Cluj-Napoca, Romania; ioana.petrusan@umfcluj.ro (I.S.); tomutaioan@umfcluj.ro (I.T.); 2 Department of Dermopharmacy and Cosmetics, Faculty of Pharmacy, “Iuliu Hațieganu” University of Medicine and Pharmacy, 400012 Cluj-Napoca, Romania; catalina.bogdan@umfcluj.ro; 3 Department 2, Faculty of Nursing and Health Sciences, “Iuliu Hațieganu” University of Medicine and Pharmacy, 400439 Cluj-Napoca, Romania; 4 Department of Nanosciences, MEDFUTURE—Institute for Biomedical Research, “Iuliu Hatieganu” University of Medicine and Pharmacy, 400349 Cluj-Napoca, Romania; valentin.toma@umfcluj.ro (V.T.); rares.stiufiuc@umfcluj.ro (R.Ș.); 5 Department of Pharmaceutical Physics & Biophysics, Faculty of Pharmacy, “Iuliu Hatieganu” University of Medicine and Pharmacy, 400349 Cluj-Napoca, Romania

**Keywords:** self-adhesive film, antiallergic, design of experiments, sustained release

## Abstract

This study aimed to design self-adhesive cutaneous films with an antiallergic effect using a Design of Experiments approach. The active pharmaceutical ingredient (API) was diphenhydramine hydrochloride (DPH). A full factorial experimental design with three factors and two levels was created. The factors were the polyvinyl alcohol (PVA) ratio, the polyacrylic acid (PAA) ratio, and the type of plasticizer. The responses evaluated were hardness, deformation at hardness, adhesive force, and in vitro DPH release profile. Eleven formulations were generated, prepared in two steps via solvent casting, and characterized in terms of mechanical and adhesive properties, as well as the in vitro DPH release profile. The PVA ratio had the most significant impact on the responses, followed by PEG 400 and PEG 4000. Four film formulations were investigated using Raman spectroscopy, which revealed that the API was distributed in both the base and adhesive layers. Consequently, an optimal formulation was prepared and characterized. Good mechanical properties (a hardness of 463.7 g and a deformation at hardness of 16.56 mm) and an increased adhesive force (76 g) were observed, while the DPH was released up to 68% over 12 h. In conclusion, a novel self-adhesive film was developed, which may enhance patients’ adherence to local antiallergic treatment.

## 1. Introduction

Papular urticaria, also known as insect bite-induced hypersensitivity, is an allergic reaction of the skin usually caused by insect bites. It frequently occurs during summer, affecting mainly children 2–10 years of age [1]. Although it is a self-limiting condition, pruritus can be very unpleasant. Its symptomatic treatment includes antipruritic agents (e.g., menthol, calamine), topical steroids, and topical and systemic antihistamines [1,2]. Topical antihistamines are typically presented as gels, creams, or ointments, which need to be applied multiple times a day, as they can be easily removed through sweating, washing, or rubbing against clothes, thereby reducing their effectiveness and requiring frequent reapplication. Therefore, a drug delivery system providing a sustained release of the active pharmaceutical ingredient (API) to the skin could be a better alternative to traditional dosage forms. An example of this is cutaneous films (patches), which are emerging dosage forms with high clinical potential. They are highly flexible, easily applied on the skin, and provide an aesthetic aspect due to their transparency [3].

To the best of our knowledge, there are currently no commercially available patches containing topical antihistamines. On one hand, there are transdermic patches with systemic antiallergic effects like Allergy Plus^®^, which includes, among others, vitamin C, quercetin, stinging nettle, luteolin, and rutin [4], or the transdermic emedastine patch, which reached phase III of a clinical trial [5]. On the other hand, there are patches designed to be applied on the insect bite (e.g., NATPAT^®^ or Moskinto^®^), but these do not contain an active pharmaceutical ingredient (API) and act through a mechanical effect [6,7]. Therefore, in this study we aimed to obtain a product with a local prolonged antiallergic effect of the API.

To reach this goal optimally, the Design of Experiments (DoE) method was used. DoE is a structured and systematic approach that allows researchers to better understand the relationship between independent variables (formulation factors) and dependent variables (responses). Compared to the “one factor at a time” approach, it is more efficient because it facilitates obtaining an optimal formulation with minimal effort, time, and developmental cost [8]. Researchers across the world have successfully applied DoE to develop topical polymeric films, such as de-esterified low-methoxyl mango-peel pectin film loaded with clindamycin for treating skin infections [9], propolis-loaded in situ film-forming systems (FFS) based on acrylic acid derivatives for surgical excision wounds [10], betamethasone 17-valerate FFS for the topical treatment of skin diseases [11], or pregabalin FFS for treating postherpetic neuralgia, to mention but a few [12].

**Figure 1:**
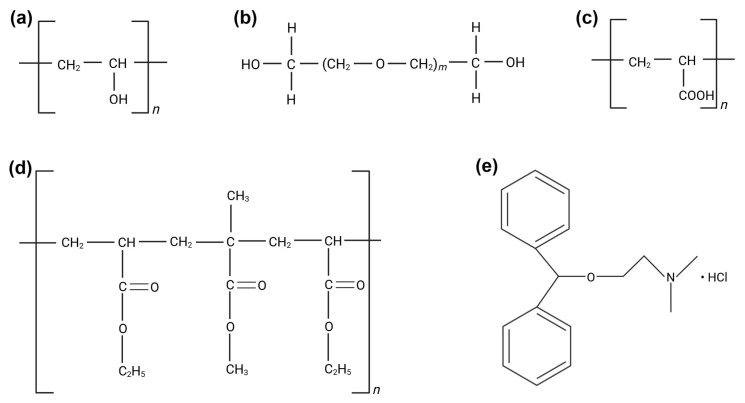
Chemical structures of (**a**) PVA, (**b**) PEG 400 (m = 8.7) and PEG 4000 (m = 69.0–84.0), (**c**) PAA, (**d**) Eudragit NM 30D, (**e**) DPH. (Figure created in Biorender. Ioana Savencu. (2025) https://app.biorender.com/illustrations/68503671b4ec7e246cb085f1).

Eudragit NM 30D (Figure 1d), a widely used polymethacrylate copolymer, was chosen in the present study due to its excellent film-forming properties and ability to modulate drug release [13,14]. It was combined with polyvinyl alcohol (PVA) (Figure 1a), due to its additional film-forming capacity and adhesive properties, strength, biocompatibility, and versatility [15].

Polyethylene glycol 400 (PEG 400) and Polyethylene glycol 4000 (PEG 4000) (Figure 1b) were used as plasticizers to improve the flexibility and permeability of the films [16], while polyacrylic acid (PAA) (Figure 1c) was chosen due to its known adhesive properties on skin and mucous membranes [17,18]. Regarding the API used in this study, diphenhydramine hydrochloride (DPH) (Figure 1e) was considered due to its wide use in relieving allergic skin reactions, including those caused by insect bites, hives, and rashes [19]. It is an antagonist of H1 (Histamine 1) receptors and, when applied topically, it decreases the histamine-mediated vascular permeability of capillaries, causing vasoconstriction and thus decreasing the flushing related to allergic reactions [20]. DPH is a first-generation antihistamine, belonging to Class I of the Biopharmaceutics Classification System (BCS I), with high solubility and high permeability [21]. Its sustained topical delivery through the polymeric matrix could provide targeted relief, reducing the need to reapply the product more times a day. However, prolonging the release of a BCS I substance is a challenge, due to its rapid dissolution and absorption. DPH was explored by Vaezi Moghaddam et al. due to its local anesthetic properties, with the potential to treat aphthous ulcers, by including the API in mucoadhesive mesoporous silica particles functionalized with amino and thiol groups [22]. Another study reported dimenhydrinate (a complex of 8-chloroteophylline and diphenhydramine) buccal mucoadhesive films to prevent and treat motion sickness [23].

This study aimed to design self-adhesive cutaneous films with prolonged antiallergic local effect, to relieve the pruritus generated by papular urticaria, using a DoE approach. According to the available data, a variety of topical/transdermal formulations based on different sorts of Eudragit have been previously reported [11,24,25,26,27], but only one of those was based on Eudragit NM 30D [28]. The novelty of this research also lies in the bi-layered structure of the medicated film, which incorporates an antihistamine, and is to the best of our knowledge proposed for the first time in the context of topical formulations with antiallergic effects. This formulation is advantageous because it separates the functions of each layer, providing enhanced adhesiveness to the upper adhesive layer, which will be directly applied to the skin, thereby ensuring good contact. In contrast, the base API-loaded layer prolongs drug release due to the extended-release properties of the chosen polymers.

## 2. Materials and Methods

### 2.1. Materials

Polyvinyl alcohol (PVA) 87–90% hydrolyzed with an average molecular weight of 30,000–70,000 (Lot number: SLCM5091), polyacrylic acid (PAA) with a molar mass of 102.13 g/mol (Lot number: SLCL8130), and diphenhydramine hydrochloride (DPH) (Lot: number WXBD5792V) were purchased from Sigma Aldrich (St. Louis, MO, USA). Polyethylene glycol (PEG) 400 was purchased from Thermo Scientific (Lot number: A0450337, Geel, Belgium), while PEG 4000 was obtained from Merck (Lot number: S6660090609, Darmstadt, Germany). Eudragit NM 30D was a kind gift from Evonik (Lot number: C230362001, Darmstadt, Germany).

To prepare the substrate needed for measuring the adhesive force, gelatin from bovine skin (Type B, gel strength ~225 g Bloom) was purchased from Sigma Aldrich (Lot number: SLCM2104, St. Louis, MO, USA), while glycerol was acquired from the International Laboratory (Lot number: IL0320221001F, Cluj-Napoca, Romania). The water used during all experiments was deionized water prepared in our laboratory.

The 85% ortho-Phosphoric acid used for preparing the aqueous HPLC mobile phase was purchased from Chemical Company (Lot number: 53, Iași, Romania), the water used was double-distilled water prepared in our laboratory, and HPLC-grade acetonitrile was procured from Promochem (Lot number: 112093, Wesel, Germany).

### 2.2. Film Preparation

Films were prepared using the solvent casting method in two steps. Firstly, a dispersion consisting of Eudragit NM 30D, 5% PVA (m/V, aqueous solution) in ratios according to each run order of the experimental design, and DPH (constant amount of 0.1 g/formulation/dish) was poured on glass Petri dishes with 9 cm diameter, 8 g of dispersion/dish, and eventually dried in ambient air for 72 h. The second step involved pouring the adhesive solution, a mixture of PAA (20% m/V, aqueous solution) and PEG 400 or PEG 4000 (the plasticizer comprising 30% of the PAA amount), onto the previously dried base layer. The volume of the adhesive solution poured into a Petri dish was either 5 mL, 7.5 mL, or 10 mL, as specified in the experimental design. After this step, the films were allowed to dry for an additional 72 h in ambient air. They were then carefully peeled off and stored in a desiccator for further experiments.

### 2.3. DoE Approach

Regarding the DoE approach, a full factorial experimental design with three factors and two levels was created using the Modde 13.0 software (Umetrics, Umeå, Sweden). The factors were the PVA ratio (the percentage of the dry base layer), the PAA ratio (the percentage of the dry film), and the type of plasticizer. Their variation levels, as well as the experimental design matrix, are presented in Table 1. The responses were hardness, deformation at hardness, adhesive force, and in vitro API release profile. Eleven formulations (N1-N11, representing eight design runs and three center points) were generated by the software, prepared, and characterized in terms of mechanical and adhesive properties, as well as in vitro API release profile.

### 2.4. Mechanical Characterization

The hardness and deformation at hardness were determined by the CT3 Brookfield Texture Analyzer (Brookfield Engineering Laboratories, Middleboro, MA, USA) using a puncture test as follows: samples of films with dimensions 3 × 3 cm were placed into the TA-FSF Film Support Fixture, while a slightly modified TA 39 probe was used to penetrate the film. The force required for film penetration and the deformation produced when films were completely broken were recorded. The following parameters were set: trigger load—10 g; test speed—0.2 mm/s; target value—40 mm. Measurements were performed in triplicate, and the mean ± standard deviation (SD) was reported.

### 2.5. Adhesive Behavior

The adhesive force was determined via the compression test of the CT3 Brookfield Texture Analyzer. The substrate used to mimic the skin was prepared according to a formulation proposed by Alarcon-Segovia et al., with some minor changes [29]. Briefly, 5 g of gelatin was hydrated in half of the total volume of distilled water for 15 min. Then, 2.5 g of glycerol was added, and the mixture was heated at 50 °C until the gelatin had dissolved entirely. Next, water was added until the total volume reached 75 mL, and the mixture was sonicated to remove any entrapped air bubbles. A total of 19 g, corresponding to 20 mL of dispersion, was poured onto each Petri dish, kept in the refrigerator for a few hours or overnight, and used over the following days. Circular samples of films with the same diameter as the TA-AACC36 probe (36 mm) were fixed on it with double-sided tape, and the Petri dish with the substrate was placed on the TA-BT-KIT fixture, secured with double-sided tape. Before running the experiment, the substrate was wetted with 100 µL of distilled water. Then, the probe was lowered at a speed of 1 mm/s until it made contact with the substrate. The trigger load was 10 g, and the target value was 100 g. After a hold time of 30 s, during which the sample remained in contact with the substrate, the probe returned to its initial position. The force required for the detachment of the sample from the substrate was recorded as the peak adhesion force, indicating the strength of interaction between the sample and the substrate. Measurements were performed in triplicate, and the mean ± SD was reported.

### 2.6. In Vitro Drug-Release Study

The release of DPH from the films was assessed using an automated Phoenix diffusion cell system (Teledyne Hanson, Chatsworth, CA, USA). The donor and receptor compartments of the cell were separated by an Express Plus PES membrane with a diameter 25 mm and a pore size of 0.45 µm (Merck Millipore, Carrigtwohill, Ireland). The release medium was an acetate buffer solution with pH = 4.7, prepared according to the European Pharmacopoeia 11.8 [30], with slight modifications. Shortly, a 40 mM sodium acetate (Lot number: 24, Chemical Company, Iași, Romania) aqueous solution was prepared, and the pH was adjusted with glacial acetic acid (Lot number: IL0420221143A, International Laboratory, Cluj-Napoca, Romania) until it reached 4.7. This pH value was chosen considering the skin surface pH, which is below 5 and on average 4.7 [31].

To ensure that the medium temperature is maintained at 32 °C during the test, the temperature of the source block and cell block was set to 32.5 °C. The mixer speed was set to 200 rpm, and the membranes were allowed to saturate with the medium and reach the desired temperature for 30 min. Small disks with diameters of 9 mm were randomly cut from the film and carefully placed with the adhesive layer in contact with the membrane situated on the lower part of the cell cap. The upper part of the cell cap was then placed on the film, fixing it tightly. Next, a glass cover was put on top of the cell cap to prevent evaporation. The released amount of DPH was determined by withdrawing 0.20 mL (0.05 mL rinse volume + 0.15 mL collected volume) of medium at predefined time points (15, 30, 45 min, 1–8 h, and 12 h) via an autosampler. The same volume of fresh medium was immediately replaced for the withdrawn volume. The collected volumes were injected into HPLC vials, which were later analyzed by HPLC (Agilent, Santa Clara, CA, USA) using a RP-HPLC method, which fulfills the following parameters listed in the ICH Q2(R2) Guideline on the validation of analytical procedures: specificity (by retention time), range, linearity within the range, and accuracy [32]. The chromatographic column was Luna^®^ 5 μm C18(2) 100 Å, 150 × 4.6 mm (Phenomenex, Torrance, CA, USA), while the mobile phase was a mixture of Phosphoric acid 0.1%–acetonitrile = 67:33 (*v*:*v*) with the UV detector set at λ = 215 nm and the fluorescence detector set at λexcitation = 230 nm and λemission = 290 nm. The cumulative release (%) of DPH was plotted against time, using the fluorescence calibration curve of pure DPH (y = 464.85x + 962.02, R^2^ = 0.9997, linear range 2–150 µg/mL). Measurements were performed in triplicate, and the mean ± SD was reported.

### 2.7. Drug-Release Kinetics

The drug-release kinetic model was established using the SigmaPlot 11.0 software (Systat Software Inc., San Jose, CA, USA) and Microsoft Excel (Microsoft Corporation, Redmond, WA, USA). Parameters such as coefficient of determination (R^2^), kinetic constant (k), diffusional release exponent (n), and Akaike Information Criterion (AIC) were calculated for the following kinetic models: Baker–Lonsdale, Korsmeyer–Peppas, Hixson–Crowell, Higuchi, first-order, and zero-order kinetics. The model with the smallest AIC value was considered the best fit for the in vitro release profile of DPH.

### 2.8. Vibrational Spectroscopic Film Characterization

Raman spectroscopy was used to investigate the distribution of APIs in the film, both within the base layer and the adhesive layer, across four film formulations containing varying amounts of each component (N2, N4, N6, N8). Measurements were conducted in two steps: initially, the pure components (DPH, Eudragit NM 30D, PVA, PAA, PEG 400, PEG 4000) were analyzed, followed by the spectroscopic analysis of the physical mixtures corresponding to each formulation (N2, N4, N6, N8). The samples were mounted on aluminum foil, which had been previously glued to microscope glass slides. The aluminum foils were washed and degreased with ethyl alcohol and ultrapure water. Powders were analyzed as such, after small amounts were deposited onto the sample holder. For samples in liquid suspension, 2 µL were deposited and left to dry on the sample holder. Measurements were taken only after the sample had been completely dehydrated. The spectra were recorded from regions located no more than 40 µm from the outer edge of the dried sample area on the aluminum foil. A Renishaw inVia Reflex confocal multilaser spectrometer with a resolution of 0.5 cm^−1^ (Renishaw™, Wotton-under-Edge, UK) was used. The excitation laser line employed in this study had a wavelength of 785 nm. The laser power (measured at the sample surface) was 113 mW (100% of the laser’s output). A 50× objective with a numerical aperture (NA) of 0.75 was used. The total signal acquisition time for one measurement was 5 s. For each sample, spectral maps were generated from at least 50 measurements taken at 50 different points. The resulting spectra were averaged to obtain a characteristic spectrum for each sample. The spectra were pre-processed by removing the influence of ambient cosmic radiation, technical noise, and smoothing the spectral slopes. This was followed by fluorescence background elimination and final spectral averaging. The final spectra were processed and visualized using OriginPro^®^ 2019 software.

In the second step, Raman measurements were performed on the four film formulations. Film pieces (~3 × 3 mm^2^ in size) were mounted perpendicularly on the sample holder to allow for cross-sectional linear spectral mapping across the film thickness. This setup enabled us to record the Raman spectra of each layer of the film samples. Measurements were performed at 1 µm space intervals. As with the precursor samples, the Renishaw inVia Reflex confocal multilaser spectrometer was used for spectral acquisitions using the same experimental conditions. The use of a 20× objective with a numerical aperture of 0.40 enabled spectral mapping across the full film thickness. Spectral pre-processing was performed using Wire 4.2 software, while the final 3D spectral representation was created using OriginPro^®^ 2019. The 3D mapping helped identify the composition, as well as the approximate thickness of each film layer, by comparing its spectrum within the 3D map to the 2D Raman spectra previously recorded for pure components.

### 2.9. Statistical Analysis

The experimental data from the DoE were analyzed by ANOVA (Analysis of Variance) using the Modde 13.0 software. *p* values below 0.05 were considered statistically significant.

## 3. Results

### 3.1. DoE Approach

**Figure 2:**
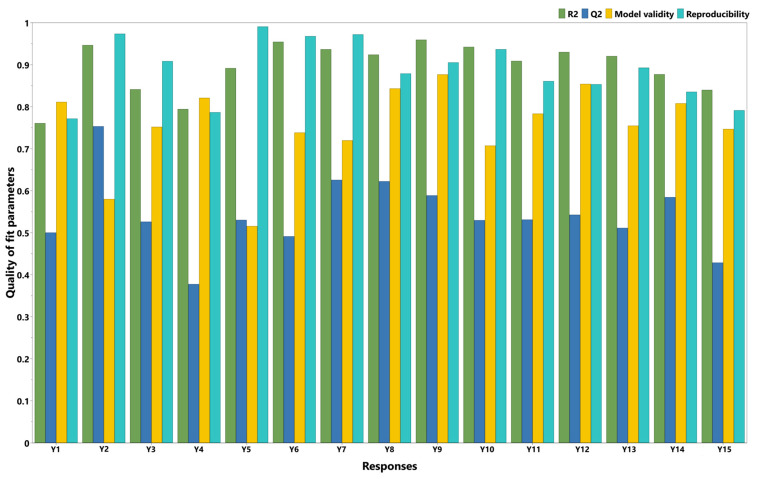
Summary of fit plot (Y1 = hardness (g); Y2 = deformation at hardness (mm); Y3 = adhesive force (g); Y4 = in vitro release 15 min (%); Y5 = in vitro release 30 min (%); Y6 = in vitro release 45 min (%); Y7 = in vitro release 1 h (%); Y8 = in vitro release 2 h (%); Y9 = in vitro release 3 h (%); Y10 = in vitro release 4 h (%); Y11 = in vitro release 5 h (%); Y12 = in vitro release 6 h (%); Y13 = in vitro release 7 h (%); Y14 = in vitro release 8 h (%); Y15 = in vitro release 12 h (%)).

The DoE approach was used to better understand the relationship between the factors and the responses by making planned changes to the factors according to a prespecified design. Initial data fitting is crucial for identifying significant factors and interactions, as well as for developing reliable predictive models for formulation optimization. In the present work, the experimental results were introduced in the design matrix (Table 2) and their fitting was carried out using the Partial Least Squares (PLS) method. The statistical parameters were R2, Q2, model validity, and reproducibility, and are displayed as histograms for each response in Figure 2. R2 is an indicator of the goodness of fit; therefore, values close to 1 imply that the model fits the data very closely. Q2 estimates the model’s predictive capacity. Values of Q2 greater than 0.5 indicate a good model, while values greater than 0.9 indicate an excellent model. The model validity value should be higher than 0.25, meaning that the model error is not significantly higher than the pure error. Finally, reproducibility represents the variation in the response under the same conditions. A value of 1 indicates perfect reproducibility, while values lower than 0.5 suggest significant errors within the model [33].

**Figure 3:**
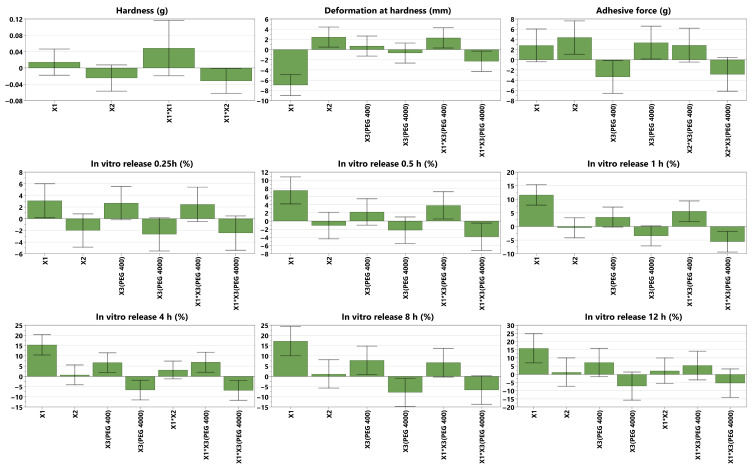
Scaled and centered coefficient plots of the mechanical properties (hardness and deformation at hardness), adhesive force and in vitro DPH release profiles for 0.25 h, 0.5 h, 1 h, 4 h, 8 h, 12 h); X1 = PVA ratio, X2 = PAA ratio, X3 = plasticizer (Figure S1 visualizes the coefficient plots for all 15 responses).

Each response was expressed mathematically as a polynomial equation that describes the response relation with each of the formulation factors:

(1) $$Y_{n}=a_{0}+a_{1}X_{1}+a_{2}X_{2}+a_{3}X_{3}+a_{4}X_{1}X_{2}+a_{5}X_{1}X_{3}+a_{6}X_{2}X_{3}+a_{7}X_{1}X_{2}X_{3}$$

where $Y_{n}$ is the response, $a_{0}$ is the mean value of the response, and $a_{1-7}$ are the regression coefficients marking the direction and the magnitude of the effect [34]. Positive or negative regression coefficients indicate a positive or a negative influence of the factors on the responses and are presented as histograms in Figure 3. They are displayed as scaled and centered coefficients, representing the coefficients of the fitted model, for which the factors were centered and scaled [33]. The significance of the effect of factors is indicated on the histograms by the confidence intervals (CIs), expressed as vertical bars. Statistically significant terms are those with CIs that do not cross the OX axis, and the smaller their heights compared to the heights of the histograms, the higher their significance.

The mechanical properties are expressed by two parameters: hardness and deformation at hardness, which had values between 232.5 and 435 g and 13.71–39.93 mm, respectively. The adhesive properties, as measured by the adhesive force, were suitable for application on the skin, with registered values ranging from 43.7 to 76.5 g.

Regarding data fitting, it was revealed that deformation at hardness best fit the model (R2 = 0.95, Q2 = 0.75), followed by adhesive force (R2 = 0.84, Q2 = 0.53) and hardness (R2 = 0.76, Q2 = 0.50). Good model validity, with values above 0.58, was observed for all responses, as well as good reproducibility, with values above 0.77. In terms of in vitro DPH release, a good fit with the model was observed for all the time points (R2 values between 0.79 and 0.96). The prediction capacity of the model described by Q2 was also good, with Q2 values above 0.5 for all time points, except for two time points during the first hour (15 and 45 min) and at 12 h. For all the time points, good model validity (above 0.51) and reproducibility (above 0.79) were observed.

The *p* values of the ANOVA regression were lower than 0.05, except for hardness (*p* = 0.081) and in vitro DPH release at 15 min (*p* = 0.057), whereas all the *p* values of the ANOVA lack of fit were higher than 0.08. These results demonstrate that the factors have a significant influence on responses and that the model exhibits no lack of fit.

### 3.2. In Vitro Drug Release

**Figure 4:**
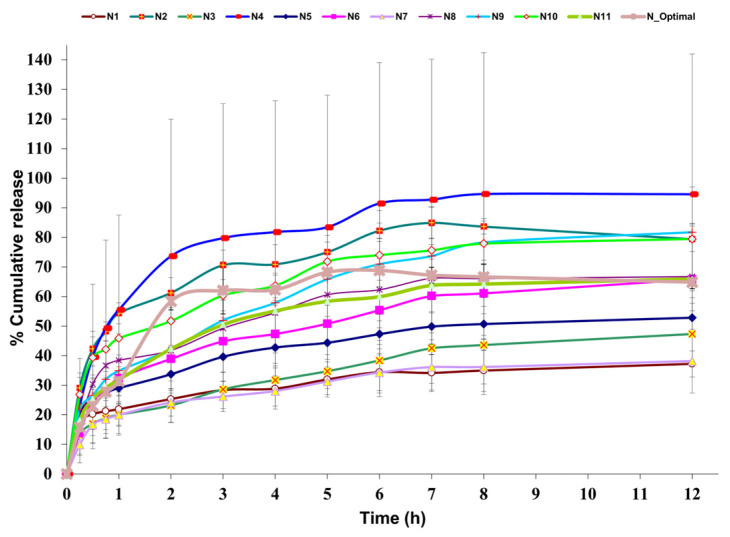
In vitro DPH release profile for 12 h (n = 3, mean ± SD), with collecting time points at 15 min, 30 min, 45 min, 1 h, 2 h, 3 h, 4 h, 5 h, 6 h, 7 h, 8h, 12 h; N1–N11 = DoE formulations, N_Optimal = optimal formulation.

The cumulative in vitro DPH release (%) from the experimental design formulations (N1–N11) as well as from the optimal formulation (N_Optimal) is depicted in Figure 4. It could be observed that N4 had the highest DPH cumulative release with a maximum of 94.5% at 12 h, while N7 displayed the lowest DPH cumulative release with a maximum of 38.1% at 12 h.

The kinetic model data of the drug-release profile are presented in Table 3. It was revealed that the in vitro DPH release profile best fitted the Korsmeyer–Peppas kinetics, due to the smallest AIC values obtained for this model across most of the formulations.

### 3.3. Spectroscopic Characterization

Raman analysis is a well-established and widely used spectroscopic technique for studying materials in various aggregate states, including solids and gels. As a non-invasive and non-destructive method, this technique was successfully used in the present study to obtain the molecular fingerprint of both precursors and final self-adhesive polymeric films.

**Figure 5:**
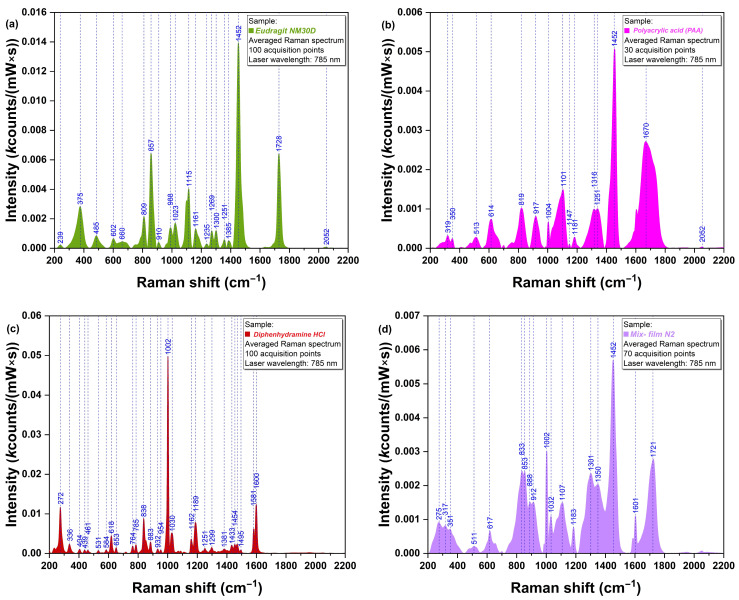
Raman spectra of (**a**) Eudragit NM 30D, (**b**) PAA, (**c**) DPH, and (**d**) physical mixture corresponding to N2 film formulation.

The spectra of pure Eudragit NM 30D (a) and pure PAA (b), which are the two main components of the base layer and adhesive layer, respectively, are presented in Figure 5. In both cases, it was revealed that their strongest peaks were located at 1452 cm^−1^, corresponding to vibrations of methylene functional groups present in their structure [35]. Pure DPH (Figure 5c) exhibited a strong peak at 1002 cm^−1^, corresponding to the ether group [36] and C-C aromatic ring vibration [35], and several moderate peaks: at 272, 838, 1030, 1189, 1581, and 1600 cm^−1^. Figure 5d presents the spectrum of the N2 physical mixture, where the strongest specific peaks of Eudragit NM 30 D, PAA (1452 cm^−1^), and DPH (1002 cm^−1^) could be observed.

**Figure 6:**
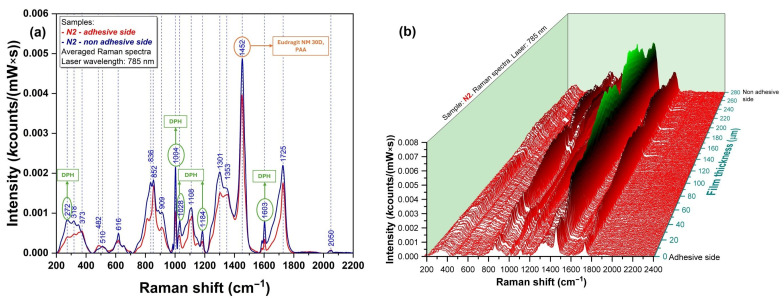
Raman spectra of N2 film formulation represented as (**a**) 2D graph; (**b**) 3D map.

To assess the API distribution in the film structure, Raman analysis of the cross-section was performed. Figure 6a presents the overlapped Raman spectra registered at the surface of the base layer and adhesive layer, respectively. It can be observed that the specific peaks of DPH (272, 1004, and 1603 cm^−1^) are present in blue (base layer) and red (adhesive layer) spectra. To better visualize API distribution within the two layers, 3D mapping was also performed. As shown in Figure 6b, the presence of DPH has been observed in both the base and adhesive layers, with higher intensities in the base layer.

### 3.4. Formulation Optimization

Based on the results mentioned above, the formulation was optimized considering that the product should be both firm and flexible for a simple manipulation, sufficiently self-adhesive to be easily applied on the skin [37], and should provide a prolonged release of the API. Therefore, the following conditions and values were set in the software: the maximization of hardness with a minimum of 330 g (target value of 420 g), a target deformation at a hardness of 30 mm, and a target value of 55 g for the adhesive force. Regarding the DPH release profile, desired target values of 40%, 60%, 80% and 85% were attributed to the time points of 1 h, 4 h, 8 h, and 12 h, respectively. The set values were chosen based on scientific literature reports and previous experience.

The Modde software generated the composition of the optimal film, as well as the predicted values, as seen in Table 4, where the experimental data was also included (observed value). The optimal formulation was prepared considering the ratios in Table 4, by the same methodology previously described in the Section 2, specifically the Section 2.2.

**Figure 7:**
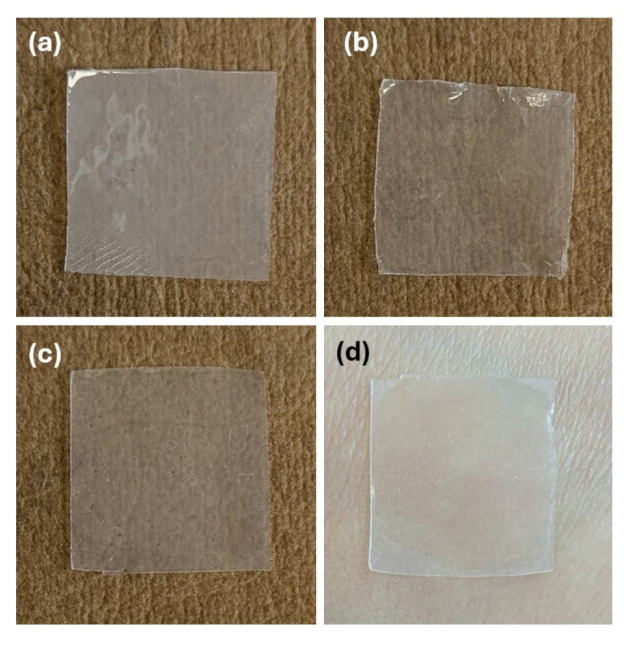
Samples of film formulations: (**a**) N2, (**b**) N6, (**c**) N_Optimal, (**d**) N_Optimal applied on human skin.

Films with high adhesive force were obtained, which were also resistant and flexible enough to ensure easy application to the skin. The in vitro DPH release over 12 h revealed that more than 50% of the drug amount was released during the first 2 h, reaching a maximum of about 69% after 6 h. It also best fit the Korsmeyer–Peppas kinetics, with R^2^ = 0.8305, k = 38.821, n = 0.2466, and AIC = 96.42. The optimal formulation is depicted in Figure 7c,d, along with two other film formulations from the DoE, N2, and N6 (Figure 7a and Figure 7b, respectively).

## 4. Discussion

After data fitting (with quality-of-fit parameters presented as histograms in Figure 2), the factors’ impact on responses could be observed and are displayed as coefficient plots in Figure 3. The obtained films were transparent to slightly white, flexible, with a smooth, shiny texture, and had a thickness ranging from 200 to 450 nm. Their physical properties varied across the DoE; that is, they were either easily detached from the Petri dish and applied on the skin without difficulty, or very adherent to the Petri dish and sticky, breaking when detached from it.

### 4.1. Mechanical Characterization

The hardness is a measure of the film’s strength. It is expressed as the maximum load (g) necessary to obtain a certain deformation [38]; therefore, the higher the value, the harder the sample [39]. In the present study, hardness was negatively influenced by the interaction between the PVA ratio and PAA ratio. The hydroxyl groups within PVA can interact with the carboxyl groups within PAA, forming intermolecular hydrogen bonds with each other. This could reduce the interactions inside each polymer network, weakening the overall film matrix [40]. No clear correlation was observed between the individual formulation factors and hardness. A possible explanation could be that, due to the elastic matrix formed during drying, Eudragit NM 30D may dominate the mechanical behavior of the films, thereby masking the effects of secondary polymers or plasticizers. For thin polymeric films, values ranging from 130 g [41] to those above 2600 g [42,43] have been previously reported; therefore, the 463.7 value of the optimal formulation falls within this range.

The deformation at hardness is the total downward distance (mm) the probe traveled once the trigger value was reached, down to the product puncture [38]. Its value is a measure of the sample’s flexibility; therefore, the longer the distance, the more flexible the sample [44]. In the DoE, it was observed that deformation at hardness was decreased by increasing the PVA ratio, possibly due to its semicrystalline nature [45]. The crystalline regions in PVA, which are rigid and less capable of stretching, make the polymer chains become more tightly packed, thus reducing the material’s ability to deform [46]. This leads to a decrease in the film’s overall flexibility and makes it more prone to fracturing under stress, resulting in lower deformation [47]. Another explanation could be the fact that PVA possesses an inherent stiffness, thus reducing the polymer matrix’s ability to stretch [48]. Interestingly, it was also observed that PVA combined with PEG 400 increased the deformation, while its combination with PEG 4000 led to a decrease in it. On one hand, increases in the deformation/flexibility of films due to PEG 400 have been previously reported [42,49]. This could be attributed to the plasticizer positioning itself between the polymer molecules and causing the disruption of the intermolecular and intramolecular hydrogen bonds, thus increasing the film’s flexibility [50]. On the other hand, PVA might have formed strong hydrogen bonds with PEG 4000, which led to a less flexible structure [51]. The PAA ratio, between 43.5% and 55.5% of dry film, positively influenced deformation at hardness. This was in accordance with the study of Faturechi et al., where increases in the PAA ratio from 10 to 30 wt% led to higher deformations [52], as well as with the study of Corona-Rivera et al., which revealed that a higher percentage of PAA led to higher elongations [53]. Various deformation values were reported in the literature. In our previous study, a deformation of 3.4 mm was recorded [42], while another study reported a value of 11.7 mm [41], closer to 16.56 mm, as in the current study’s optimal formulation.

### 4.2. Adhesive Behavior

The adhesive force is expressed as the load (g) needed to detach the probe from the sample [38]. It was essential to investigate the self-adhesive nature of the films, which should provide both easy application to the skin and facile detachment, ideally without residue. As expected, the adhesive force was positively influenced by increasing the ratio of PAA due to its mucoadhesive properties, which enable it to adhere to skin or other surfaces [54], due to electrostatic forces and hydrogen bonds [55]. PAA can form either intramolecular hydrogen bonds with itself through carboxyl group dimerization, or intermolecular hydrogen bonds with PEG [56] or the gelatin substrate used to assess adhesion. Due to its hydrophilicity, increasing the PAA ratio enhances the film’s wetting, allowing it to spread more evenly across the substrate and maximize the contact area, thereby increasing the adhesive force [18]. PEG 4000 also increased the adhesive force, possibly due to the high number of strong hydrogen bonds between the hydroxyl functional groups of the polymer and the hydroxyl or amino functional groups within the gelatin substrate. On the contrary, PEG 400 decreased the adhesive force, which is in accordance with our previous study [42], where a similar behavior was observed, possibly due to the increased free motion of the polymer resulting from the presence of the plasticizer. The adhesive force of the optimal formulation was significantly higher than that reported in previous studies, where values between 20 and 30 g were observed [42,43], thereby ensuring the self-adhesion of the film.

The appropriate mechanical strength (hardness) makes the optimal formulation easy to detach from its support and manipulate, while its good flexibility (deformation at hardness) and high adhesive force enable it to be comfortably applied on the skin, while providing an aesthetic appearance due to its transparency.

### 4.3. In Vitro Drug Release

The in vitro DPH release profile was studied because the final product was aimed to have a prolonged antiallergic local effect. The DoE revealed that the PVA ratio strongly enhanced the drug release, due to its water-absorption capacity and swelling, which created a more porous structure of the film, thus allowing the hydrosoluble API to diffuse more easily through the polymeric matrix [57]. PEG 400, either alone or combined with PVA, also increased the release, possibly due to the enhanced flexibility, which increased the permeability [58]. On the contrary, PEG 4000, either alone or combined with PVA, decreased the drug release, in accordance with the Handbook of Pharmaceutical Excipients, which states that the drug-release rate of water-soluble APIs decreases when increasing the molecular weight of PEG [16]. This could be attributed to strong hydrogen bonds, which decrease the mobility of the polymer matrix. In addition, due to the higher molecular weight, PEG 4000 dissolves more slowly than PEG 400, forming a gel that slows the diffusion of the API through the polymeric matrix. Interestingly, no significant influence of the plasticizer, whether PEG 400 or PEG 4000, was observed during the first hour, possibly due to the time required for the film to hydrate fully. However, its impact became evident between the first and eighth hours, and then it became insignificant again at 12 h. This could be attributed to matrix saturation with water over time and the potential leaching of plasticizers. To summarize, the explanations mentioned earlier demonstrate why the formulation with the maximum amount of PVA and PEG 400 (N4) displayed the highest DPH in vitro cumulative release, in contrast to the formulation without PVA but with the maximum amount of PEG 4000 as a plasticizer (N7).

The API release from the optimal formulation reached a maximum of approximately 69% at 6 h, possibly due to part of the API being entrapped in the polymeric matrix. A similar behavior of partial API release was observed by another research group, who reported Eudragit NM 30D-based films [28]. The initial DPH rapid release within the first hour (approximately 31%) can be explained by the partial distribution of the API in the adhesive layer, as supported by the Raman analysis below. The API in the adhesive layer might rapidly dissolve and diffuse through the polymeric network without any lag time. This behavior was similar to the findings of Rao et al., where an initial burst effect of propranolol hydrochloride within transdermal polymeric films was observed [59]. However, this phenomenon could benefit the patient by soothing histamine-mediated pruritus, while the following sustained release could be advantageous because it ensures a prolonged therapeutic effect.

According to the lowest AIC value, the formulations in the DoE (N1-N11, except N4 and N9, which fitted best with the Baker–Lonsdale model), as well as the optimal formulation (N_Optimal), were revealed to best suit the Korsmeyer–Peppas model. Additionally, the lowest obtained values for AIC correlate with the highest R^2^ values, which, in ideal cases, should be 1. In the present study, all the R^2^ values within the DoE corresponding to the Korsmeyer–Peppas model were higher than 0.92 (Table 3), close to those reported by Kim et al., which ranged from 0.8936 to 0.9499 [60], or by Sampaopan et al., with R^2^ values ranging from 0.9636 to 0.9670 [28]. According to Korsmeyer et al., regarding kinetic parameters, if the value of n equals 0.5, the drug release is controlled by the Fickian diffusion, due to the concentration gradient, through the polymeric matrix [61]. The n values of the present study, ranging from 0.193 to 0.329, were similar to those reported by Sampaopan et al., ranging from 0.2658 to 0.3142 [28]. Other researchers also reported n values below 0.5 [62,63], indicating quasi-Fickian diffusion (partial diffusion) [63]. In the present study, the drug-release mechanisms, expressed as Fickian or quasi-Fickian diffusion, were somewhat expected, as a hydrophilic drug incorporated in a matrix is primarily released by diffusion [64]. However, we wanted to verify if other physical phenomena, explained by different models, occurred during the testing. For example, the Baker–Lonsdale model is used to describe the drug-release kinetics from spherical polymeric matrices governed by Fick’s law [65], and N4 and N9 were revealed to best fit this model. The Hixson–Crowell model helps describe drug release from formulations where the surface area is reduced proportionally due to erosion [64]. It was assumed that such an event might occur because of the hydrophilic matrices consisting of PVA and PAA, which could erode in an aqueous release medium.

### 4.4. Spectroscopic Characterization

The 3D vibrational map of the film’s cross-section was the most representative of illustrating how the API was distributed in both the base and adhesive layers. It revealed a higher proportion of API in the base layer, as expected, since only the base-layer dispersion contained DPH. The migration of DPH in the adhesive layer can be attributed to the partial dissolution of DPH in water during the pouring of the adhesive aqueous solution (PAA + plasticizer). This phenomenon, confirmed by Raman analysis, was beneficial because it facilitated a partial and faster DPH release in the initial part of the in vitro drug-release study (the adhesive layer was in direct contact with the membrane), thereby ensuring both a rapid and prolonged antiallergic effect. Raman spectroscopy has also been successfully employed for assessing the distribution of DPH in pharmaceutical wafers. Haag et al. [66] had shown that the dominant peak (1002 cm^−1^) can be successfully used for quantifying DPH in the wafers. The same characteristic band has been employed by Orkoula et al. [67] to determine the presence of DPH in the liquid formulation.

## 5. Conclusions

Eleven formulations were successfully prepared according to the DoE and characterized in terms of mechanical and adhesive properties, and in vitro DPH release profile. The adhesive force increased with the PAA ratio and PEG 4000 and decreased with PEG 400. The deformation at hardness was positively influenced by PAA ratio, while a substantial negative impact of PVA ratio was observed on it. The in vitro DPH release was strongly positively impacted by increasing the PVA ratio, followed by a positive influence of PEG 400 and a negative influence of PEG 4000. In contrast, no impact of the PAA ratio was observed. Raman spectroscopy revealed that the API was distributed in both the base and adhesive layers, although during the preparation step, only the base-layer dispersion contained the API. The optimal formulation exhibited a high adhesive force of 76 g, along with good mechanical properties (a hardness of 463.7 g and a deformation of 16.56 mm), thereby providing self-adhesiveness, sufficient hardness, and flexibility, allowing the film to be easily applied to the skin. The sustained release of the API could enable its application once or twice a day, thus improving the patient’s adherence to antiallergic local treatment. However, to confirm this, further in vivo studies are required. Animal models could be used to understand pharmacokinetic and toxicological profiles, while human trials could assess efficacy, safety, and therapeutic potential under authentic physiological conditions.

## Figures and Tables

**Table 1:** The factors of the DoE with their variation levels and the experimental design matrix.

Factors	Symbol	Type	Levels		
PVA ratio (%)	X1	Quantitative	0	15	30
PAA ratio (%)	X2	Quantitative	43.5	49.5	55.5
Plasticizer	X3	Qualitative	PEG 400, PEG 4000		
**Exp Name**	**Run Order**	**PVA ratio (X1) (%)**	**PAA ratio (X2) (%)**		**Plasticizer (X3)**
N1	5	0	43.5		PEG 400
N2	3	30	43.5		PEG 400
N3	4	0	55.5		PEG 400
N4	8	30	55.5		PEG 400
N5	6	0	43.5		PEG 4000
N6	2	30	43.5		PEG 4000
N7	1	0	55.5		PEG 4000
N8	9	30	55.5		PEG 4000
N9	7	15	49.5		PEG 400
N10	11	15	49.5		PEG 400
N11	10	15	49.5		PEG 400

**Table 2:** A matrix of the results.

Exp	Y1	Y2	Y3	Y4	Y5	Y6	Y7	Y8	Y9	Y10	Y11	Y12	Y13	Y14	Y15
N1	321.8	31.94	43.7	17.40	20.14	21.19	21.88	25.29	28.33	28.82	31.93	34.39	34.12	34.93	37.20
N2	425.7	19.19	46.8	29.05	42.27	48.19	54.31	61.22	70.59	70.85	74.98	82.24	84.91	83.63	79.39
N3	393.8	37.53	51.8	13.17	16.96	18.84	20.01	23.16	28.55	31.70	34.69	38.33	42.44	43.54	47.26
N4	305.2	26.07	66.8	22.88	39.53	49.29	55.46	73.60	79.70	81.74	83.38	91.43	92.70	94.60	94.52
N5	359.7	39.64	58.0	19.98	25.41	27.56	29.02	33.71	39.62	42.74	44.36	47.28	49.82	50.70	52.83
N6	435.0	13.71	58.2	13.83	24.75	27.89	32.39	38.89	44.81	47.31	50.79	55.34	60.15	61.06	65.80
N7	321.5	39.93	57.8	9.87	16.65	18.51	19.92	24.14	26.15	28.01	31.20	34.26	36.10	36.14	38.12
N8	342.0	21.71	63.2	17.27	30.36	36.62	38.43	41.79	49.25	54.46	60.57	62.28	66.14	66.08	66.74
N9	332.0	31.52	55.5	21.75	26.57	31.95	34.88	42.14	51.92	57.85	65.83	70.91	73.65	78.28	81.73
N10	303.7	33.38	52.5	26.85	39.33	42.17	45.85	51.71	60.34	63.67	71.82	73.99	75.59	77.86	79.46
N11	232.5	30.68	76.5	18.17	25.41	29.17	31.87	42.27	50.46	55.07	58.33	59.87	63.72	64.15	65.88

Notes: Y1 = hardness (g); Y2 = deformation at hardness (mm); Y3 = adhesive force (g); Y4 = in vitro release 15 min (%); Y5 = in vitro release 30 min (%); Y6 = in vitro release 45 min (%); Y7 = in vitro release 1 h (%); Y8 = in vitro release 2 h (%); Y9 = in vitro release 3 h (%); Y10 = in vitro release 4 h (%); Y11 = in vitro release 5 h (%); Y12 = in vitro release 6 h (%); Y13 = in vitro release 7 h (%); Y14 = in vitro release 8 h (%); Y15 = in vitro release 12 h (%). Results are expressed as mean (n = 3).

**Table 3:** Drug-release kinetic models (N1–N11).

Formulation		N1	N2	N3	N4	N5	N6	N7	N8	N9	N10	N11
Baker–Lonsdale	R^2^	0.5012	0.8742	0.9289	0.9788	0.7011	0.9205	0.7848	0.7937	0.9811	0.8365	0.8528
	AIC	86.75	91.66	70.66	**73.76**	90.41	80.82	79.05	93.47	**68.72**	93.76	89.37
	k	0.0033	0.0368	0.0046	0.0532	0.0073	0.0112	0.0032	0.0142	0.0203	0.0255	0.0130
Korsmeyer–Peppas	R^2^	0.9913	0.9289	0.9861	0.9238	0.9804	0.9789	0.9738	0.9431	0.9812	0.9630	0.9485
	AIC	**36.13**	**86.24**	**51.41**	92.39	**57.02**	**65.56**	**53.64**	**78.73**	70.61	**76.42**	**77.72**
	k	22.770	52.705	20.622	55.212	30.189	31.229	19.826	37.635	36.613	46.246	34.923
	n	0.196	0.193	0.329	0.235	0.226	0.300	0.267	0.241	0.330	0.223	0.266
Hixson–Crowell	R^2^	0.0000	0.2805	0.3256	0.5742	0.0000	0.4267	0.0000	0.1912	0.7795	0.3784	0.3137
	AIC	104.71	114.33	99.91	112.76	109.97	106.50	101.39	111.23	100.65	111.12	109.39
	k	0.015	0.080	0.020	0.088	0.027	0.039	0.016	0.050	0.062	0.071	0.046
Higuchi	R^2^	0.3746	0.2932	0.8763	0.5318	0.5369	0.8067	0.7060	0.5775	0.8703	0.5052	0.6773
	AIC	89.69	114.10	77.86	114.00	96.11	92.37	83.10	102.79	93.74	108.15	99.57
	k	12.470	28.585	14.596	32.433	17.476	20.850	12.409	22.395	25.920	26.598	21.805
First order	R^2^	0.0000	0.5943	0.4507	0.9289	0.0000	0.5727	0.0000	0.3710	0.8476	0.5450	0.4851
	AIC	103.32	106.88	97.25	89.49	107.42	102.68	99.65	107.96	95.84	107.06	105.65
	k	0.053	0.492	0.069	0.717	0.097	0.140	0.053	0.178	0.230	0.302	0.164
Zero order	R^2^	0.0000	0.0000	0.0000	0.0000	0.0000	0.0000	0.0000	0.0000	0.0000	0.0000	0.0000
	AIC	107.42	130.74	105.31	132.43	115.39	116.65	104.75	122.03	120.79	126.89	120.36
	k	3.453	7.779	4.204	8.938	4.857	5.926	3.491	6.211	7.437	7.356	6.085

lowest values are shown in bold.

**Table 4:** Optimal formulation composition and attributes.

Factor		Symbol		Value (%)
PVA ratio		X1		23.14
PAA ratio		X2		43.75
Plasticizer		X3		PEG 400, 30% of PAA
**Response**	**Symbol**	**Predicted value**	**Observed value**	**Bias (%)**
Hardness (g)	Y1	371.51	463.7	24.81
Deformation at hardness (mm)	Y2	24.23	16.56	−31.65
Adhesive force (g)	Y3	47.77	76.00	59.10
In vitro release 0.25 h (%)	Y4	25.36	15.74	−37.93
In vitro release 0.5 h (%)	Y5	35.71	22.91	−35.84
In vitro release 0.75 h (%)	Y6	39.71	27.73	−30.17
In vitro release 1 h (%)	Y7	46.15	31.28	−32.22
In vitro release 2 h (%)	Y8	57.04	58.39	2.37
In vitro release 3 h (%)	Y9	63.77	62.02	−2.74
In vitro release 4 h (%)	Y10	65.45	62.26	−4.87
In vitro release 5 h (%)	Y11	69.76	68.19	−2.25
In vitro release 6 h (%)	Y12	75.67	68.78	−9.11
In vitro release 7 h (%)	Y13	78.22	67.16	−14.14
In vitro release 8 h (%)	Y14	80.58	66.60	−17.35
In vitro release 12 h (%)	Y15	78.75	64.83	−17.68

## Data Availability

The raw data supporting the conclusions of this article will be made available by the authors on request.

## Supplementary Materials

The following supporting information can be downloaded at: https://www.mdpi.com/article/10.3390/polym17131867/s1; Figure S1: Scaled and centered coefficient plots of the mechanical properties (hardness and deformation at hardness), adhesive force, and in vitro DPH release profile (0.25 h, 0.5 h, 0.75 h, 1 h, 2 h, 3 h, 4 h, 5 h, 6 h, 7 h, 8 h, 12 h); X1 = PVA ratio, X2 = PAA ratio, X3 = plasticizer.

## Abbreviations

The following abbreviations are used in this manuscript:

API	Active pharmaceutical ingredient
PVA	Polyvinyl alcohol
PAA	Polyacrylic acid
DPH	Diphenhydramine hydrochloride
PEG	Polyethylene glycol
DoE	Design of Experiments
HPLC	High-Performance Liquid Chromatography
FFS	Film-forming systems
CI	Confidence interval
PES	Polyethersulfone
AIC	Akaike Information Criterion
ANOVA	Analysis of Variance
PLS	Partial Least Squares
