# Supplementary figures and images for: Development and Characterization of Self-Adhesive Polymeric Films with Antiallergic Effect
Source: Polymers (Basel). 2025 Jul 3;17(13):1867. doi: 10.3390/polym17131867 (PMC12251892; doi:10.3390/polym17131867)

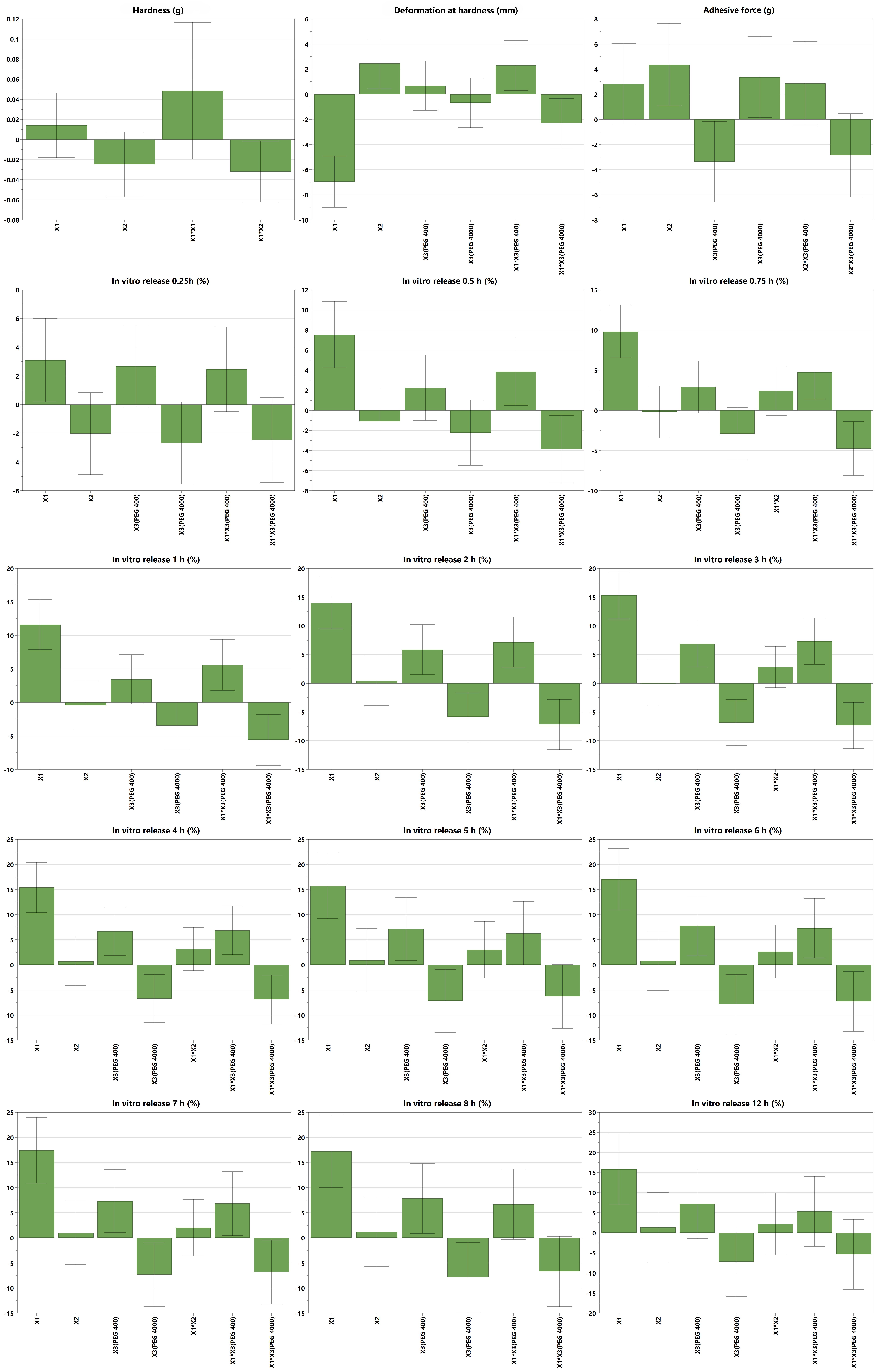

Supplement: Supplementary file 1 [file polymers-17-01867-s001.zip › polymers-3704250-supplementary.jpg]
